# The thin line between seeing risks and venturing scientific progress

**DOI:** 10.1007/s00417-022-05557-1

**Published:** 2022-02-03

**Authors:** Moritz Lindner

**Affiliations:** Institute of Physiology and Pathophysiology, Department of Neurophysiology, Philipps University, Marburg, Germany

**Keywords:** Optogenetics, Gene Therapy, Vision Restoration, Ethics, Clinical Trials

Dear Editor,

With great interest I have read the Mini-Review entitled “Restoring vision using optogenetics without being blind to the risks” recently published by Harris and Gilbert in Graefe’s Archives [1]. I highly appreciate that the authors put a spotlight onto ethical aspects of clinical trials in optogenetic vision restoration.

The authors argue by the potential risks arising for an individual from trial participation. As probably any medical progress comes at a certain risk for those volunteering as trial participants this immediately puts forward the next questions: Which risks should we be willing expose trail participants to? And, if we can’t asses a risk on the basis of animal or earlier phase clinical trial data, from what degree of theoretical plausibility on should it be considered?

Harris and Gilbert’s article teaches us how narrow the lines between the possible answers to these questions are and how careful the available evidence needs to be weighted. For instance, for immunity concerns the authors suggest that studies should not for “the sole purpose of novelty” test novel Adeno-associated virus (AAV) serotypes (and optogenetic tools). Herein, it needs to be taken into account that there is now data on safety for a variety of AAV serotypes from numerous phase I/II clinical trials that can serve as reference [3]. Experimental evidence moreover suggests that not the serotype but rather cis-regulatory elements determine immunogenicity [4]. Harris and Gilbert in particular discuss the use of novel AAV2.7m8 (employed in Phase I/II NCT03326336, Gensight [5]) given that “wild-type” AAV2 has already obtained regulatory approval. Interestingly, AAV2.7m8 has a substantially increased transduction efficacy and the success of any optogenetic therapy likely depends on the proportion of cells expressing the optogenetic tool after treatment. It is therefore probable that choosing novel AAV2.7m8 in NCT03326336 was one of the essential preconditions for obtaining detectable signs of efficacy [5].

Let us imagine regulators would request future optogenetic trails to use “clinically proven” AAV serotypes to reduce trail-related risks. This would decrease the probability of such trails to achieve detectable degrees of vision restoration. With two or three trails failed, this may lead to disregarding a possibly working therapeutic concept, thereby withholding future generation a treatment that could become the first to restore vision to a level above that of legal blindness. This exemplifies how the most precautious approach is not necessarily the most ethical.

But what if data to assess an assumed risk is not available and assessment based on theoretical considerations is required? Harris and Gilbert’s article offers an interesting starting point for further discussion. The authors argue that an optogenetic gene therapy treatment might cause a form of immunity that could induce immune responses to future AAV-based treatments. As an example, they put forward the Oxford and J&J COVID-19 vaccines. it is important to reflect that both these vaccines are Adenovirus- rather than AAV-based [2] and these viruses are genetically entirely unrelated. Hence, the theoretical chance of inducing cross-immunity is close to non-existent. The same applies for the concern that some AAV serotypes may increase the risk of contracting Human immune deficiency virus (HIV). Taking such a highly precautious standpoint and considering even unlikely risks is consequent when the sole aim is to nullify the risk for an individual trail participant. But in a broader sense, this comes at a price: COVID-19 and HIV are highly emotional topics and communicating improbable risks relating to those may insecure patients otherwise willing to participate in optogenetic trails. Moreover, as AAV are the standard shuttle in ocular gene therapy the impact of this may spread beyond optogenetics: it may plant unnecessary concerns even into the mind of patients eligible for FDA/EMA approved gene therapies.

Weighting risks in clinical tails is a thin line on complex terrain. As we continue discussing the ethics of optogenetic gene therapy trails, we should not only consider the risks we bear on (informed) trial participants, but what the price will be for not doing so.
